# Effect of Regional Anesthesia Techniques on Hemodynamic Variables Measured With FloTrac/Vigileo™ System: A Prospective Cohort Study

**DOI:** 10.7759/cureus.92589

**Published:** 2025-09-17

**Authors:** Sandeep Diwan, Suhrud Panchawagh, Parag K Sancheti, Abhijit Nair

**Affiliations:** 1 Anaesthesiology, Sancheti Institute for Orthopaedics and Rehabilitation, Pune, IND; 2 Neurology, Smt. Kashibai Navale Medical College and General Hospital, Pune, IND; 3 Orthopaedics and Trauma, Sancheti Institute for Orthopaedics and Rehabilitation, Pune, IND; 4 Anesthesiology, Ibra Hospital, Ibra, OMN

**Keywords:** lumbar epidural anesthesia, lumbosacral plexus block, proximal femur fracture, proximal femur nailing, regional anesthesia

## Abstract

Background and objectives

Neuraxial anesthesia, particularly the subarachnoid block (SAB) or spinal anesthesia, is a viable option utilized for the surgical management of proximal femoral fractures (PFF) in the geriatric population. The hemodynamic effects of SAB could be detrimental in the geriatric population, especially with cardiac comorbidities. A segmental epidural or lumbosacral plexus block (LSPB) is particularly safe for this cohort while undergoing proximal femoral nail (PFN) surgery. We decided to monitor variations in cardiac output (CO) and stroke volume (SV) in a cohort of patients who were undergoing proximal femoral nailing for PFFs. The FloTrac/Vigileo™ System was employed to monitor hemodynamic variables continuously.

Methods

This prospective, observational study included 29 patients with PFFs. Heart rate, CO, SV, and mean arterial pressure (MAP) were continuously monitored from the commencement of regional anesthesia (segmental epidural or LSPB) to the conclusion of the surgical procedure. Before regional anesthesia techniques, each patient was administered a mini-fluid challenge.

Results

Eleven patients were recruited in the epidural group, and 18 patients in the LSPB group. Compared to the epidural group, for each unit increase in SV in the LSPB group, the rate of increase in MAP was higher by 0.13 (p<0.001). Compared to the epidural group, for each unit increase in CO in the LSPB group, the rate of increase in MAP was higher by 2.78 (p<0.001). Compared to the epidural group, for each minute passed under the LSPB group, the rate of increase in CO was lower by 2.18 (p<0.001).

Compared to the epidural group, for each minute passed under the LSPB group, the rate of increase in SV was lower by 0.05 (p<0.001). In four patients in whom LSPB was administered, noradrenaline and vasopressors were given, while six patients in the epidural group received inotropes and vasopressors.

Conclusions

In patients having PFN for PFFs, regional anesthesia in the form of a segmental epidural or lumbosacral plexus provides steady hemodynamics during the perioperative phase. Furthermore, maintaining proper fluid balance can meet a fluid challenge even in high-risk populations.

## Introduction

Inotropic/vasopressor support may be necessary for elderly high-risk patients who are undergoing proximal femoral nails (PFN) for a proximal femoral fracture under a subarachnoid block (SAB) [1]. End-organ dysfunction/damage, including acute kidney injury and myocardial infarction, was documented in patients who maintained a mean arterial pressure (MAP) of less than 80 mmHg for a period exceeding 10 minutes [2]. The maintenance of hemodynamic homeostasis is significantly influenced by perioperative fluid management. In elderly hip fractures, fluid losses can be multifactorial, and the attending anesthesiologist is responsible for the vital task of intraoperative fluid management in high-risk patient groups. Additionally, the literature demonstrates that the monitoring and optimization of cardiac output (CO) will expedite the recovery process and enable early discharge [3,4]. A stroke volume (SV)-based fluid management and MAP-based inotropic intervention is demonstrated through the use of a mini-invasive cardiac monitoring technique [5]. We refrained from administering spinal anesthesia and instead selected continuous lumbar epidural (CLE) and lumbosacral plexus block (LSPB) as the primary anesthetic techniques for American Society of Anesthesiologists- physical status (ASA-PS) III and IV patients with proximal femoral fractures (PFF). CLE induces bilateral lower limb vasodilation; however, the effects of segmental sympathectomy would be less than those of spinal anesthesia [6]. Unilateral lower limb vasodilation is the result of LSPB [7]. The hemodynamic variables (SV, CO, MAP, and heart rate) were assessed directly from the FloTrac/Vigileo™ system after the implementation of these procedures. Additionally, the interventions in the form of inotropic and fluid therapy were evaluated in both groups.

The objective of this study was to assess the impact of CLE and LSPB on the hemodynamic variables. Additionally, the interventions, which included intravenous fluids and inotropes/vasopressors, were examined in both groups.

## Materials and methods

The institutional ethical committee at Sancheti Hospital, Pune, Maharashtra State, India, approved this single-center, observational study (approval date: 1st September 2020). A total of 29 patients, aged 77 to 101 years, were admitted with PFFs who were classified in ASA-PS III and IV. This study encompassed patients who were scheduled for a PFN between January 2021 and January 2022. We administered a CLE and/or an LSPB to patients with an ASA-PS III and IV following our prior experience [2]. The following were the exclusion criteria: patient refusal, coagulation system disorders, infections of the back and gluteal region, known allergies to local anesthetics, pre-existing neurological disorders, or inability to comprehend the local language. Additionally, patients with severe valve regurgitation, hepatic disease, peritonitis, and arrhythmia were eligible for exclusion.

The primary objective was to ascertain the impact of epidural and LSPB on the CO indices and SV. The secondary objective was to comprehend the fluid requirements, ionotropic support, and adverse events. All patients were medically optimized 24 hours before the surgical procedure. Patients were transferred to the recovery area of the operating room on the day of the procedure. They underwent an ultrasound-guided femoral nerve block with 1% lidocaine in a volume of 7-10 ml. In ASA 3 and 4 patients, our institution's monitoring standards include invasive arterial pressure monitoring, pulse oximetry, and a 3-lead electrocardiogram. The patients were transported to the operating room and placed on the operating table. After local infiltration of 1% lidocaine 1-2 mL), the left radial artery was cannulated (20 G/1;10 mm × 45 mm; BD Critical Care Systems Pte Ltd., Singapore). The arterial catheter was connected to a Vigileo™ monitor (software version V 1.10; Edwards Lifesciences, Irvine, CA) and the FloTrac™ sensor [8].

FloTrac/Vigileo™

The FloTrac/Vigileo™ system is a minimally invasive CO measuring device that minimizes operator error and does not necessitate calibration. It calculates CO. It integrates the actual vascular tone determined by waveform analysis and patient characteristics. The arterial waveform is analyzed for 20 seconds at a frequency of 100 Hz. The SV is determined by the average and standard deviations of a specific waveform, while the CO is determined by the heart rate and the calculated SV, which are updated every 20 seconds [1].

Baseline hemodynamic values were recorded in the supine position before the patient was administered regional anesthesia. The following data were collected: cardiac output/cardiac index, systolic and diastolic pressures, SV/SV index, and heart rate. To monitor and sustain a 10% increase in the SV of the baseline in each patient, mini-fluid challenge tests (100 ml of Ringer’s Lactate) were administered. In patients with an ejection fraction ranging from 25% to 30% and 20% to 25%, a CLE or LSPB was implemented based on the preoperative echocardiographic findings.

Under aseptic conditions, the same anesthesiologist implemented a regional anesthesia technique in the lateral decubitus position, with the operative side being non-dependent.

Continuous lumbar epidural (CLE)

**Figure 1:**
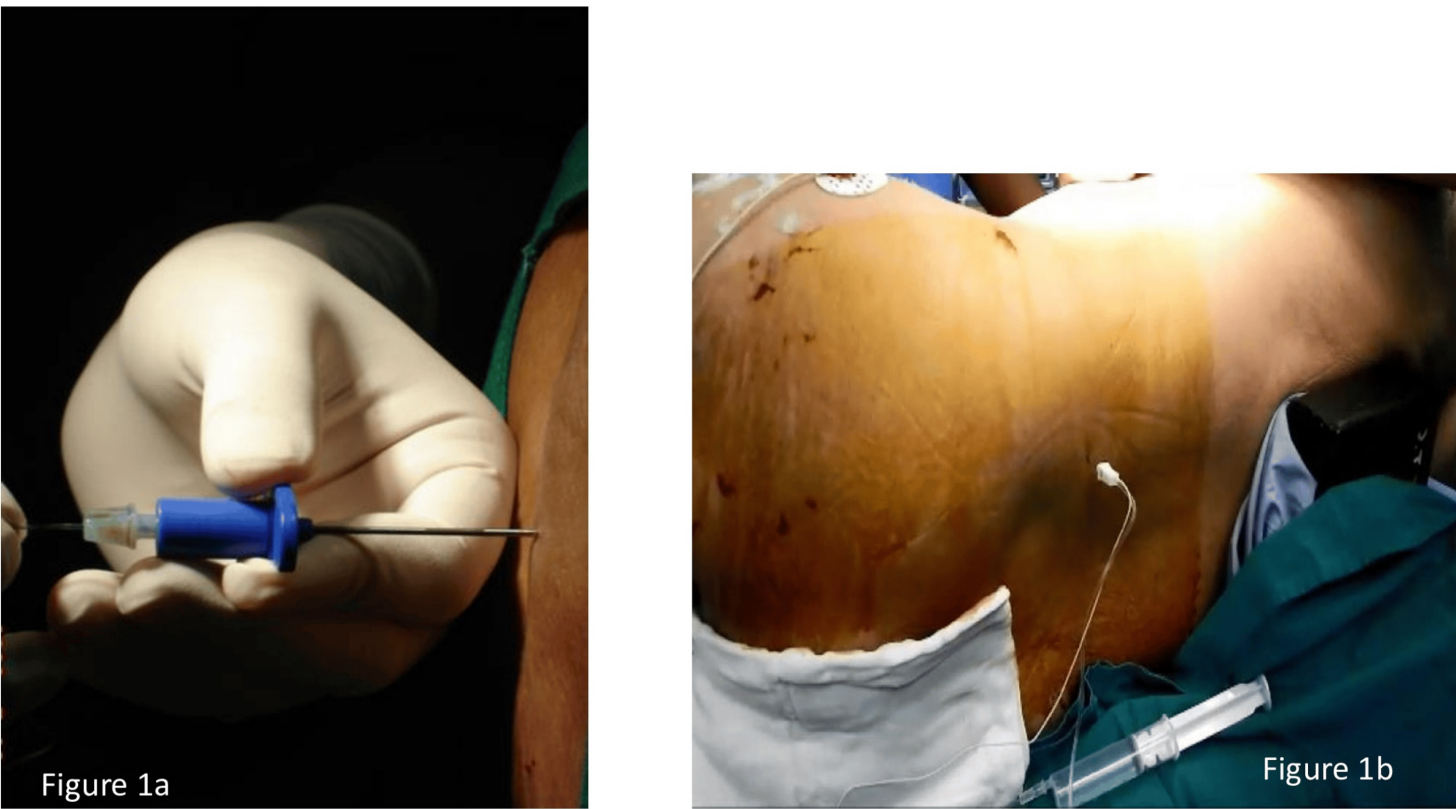
Interventions performed a) Continuous lumbar epidural; b) neurostimulation-guided lumbar plexus block

1% lidocaine was infiltrated deeply into the subcutaneous tissue at the L2-3 interspinous area. The lumbar epidural space was identified by a reduction of resistance to air using an 18-gauge Tuohy needle (Figure 1a). The catheter was inserted at a distance of 3 to 5 cm after a bolus of 5 ml of local anesthetic (LA), an equal volume of 0.5% bupivacaine and 1.5% lidocaine, respectively. The catheter was used to administer an additional bolus of 5-7 ml of LA. A light pinprick was used to assess the analgesia level, and if it was found to be insufficient, an additional bolus of 3-5ml of LA was administered. For 48 hours, all patients were administered 0.1% ropivacaine at a rate of 6 ml per hour postoperatively.

Lumbosacral plexus block (LSPB)

The lumbar plexus block (LPB) was performed using Winnie’s landmark, while the sacral plexus block (SPB) was performed using Mansour's technique. The endpoint for the LPB was defined as quadriceps contractions between 0.3 mA and 0.5 mA, while the endpoint for the SPB was either a plantar or dorsiflexion of the foot. This was achieved using a nerve stimulator (Stimuplex, HNS 11, Braun Medical, Melsungen, Germany) and stimulating needles (Stimuplex-A Needle, 100 mm/21G, Braun Medical, Melsungen, Germany) with a stimulating frequency of 1 Hz, a stimulating current of 1 mA, and a pulse duration of 0.1 ms. Figure 1b illustrates that 20-22 ml of LA (0.5% Bupivacaine and 1.5% Lignocaine) was administered to the lumbar plexus with intermittent aspiration at 5 ml/second and 10-12 ml to the sacral plexus at 0.5 ml/second after negative aspiration for blood and cerebrospinal fluid. The patient’s inability to extend the knee and plantar or dorsiflex the foot was used to determine whether an adequate block was present. A Bromage scale was applied to the dependent limb to exclude the possibility of epidural dissemination. The surgical procedures were conducted in the lateral decubitus position without the use of a fracture table. Various time points were utilized to accomplish continuous data recording. Consequently, all patients were transferred to the intensive care unit. Paracetamol was administered intravenously at a rate of 30 mg/kg every 8 hours. The administration of rescue analgesia in the form of an intravenous fentanyl infusion at a rate of 1-2 ml per hour (10 mcg/ml) was instituted if the pain score on the numerical rating scale (NRS) exceeded 4.

Statistical methods

Data are summarized as medians (interquartile range (IQR)) for continuous variables and frequencies (percentage) for categorical variables. Exploratory analyses for continuous variables are performed using histograms and scatterplots. Linear mixed-effects models (estimated using Restricted Maximum Likelihood (REML) and the nloptwrap optimizer in the “lme4” package in R), with each subject acting as a random intercept, were used to model and visualize linear relationships between CO, SV, MAP, and time. The regression model is visualized as a scatter of the predicted values and a best-fit line. 95% confidence intervals (CIs) and p-values were computed using a Wald t-distribution approximation. The regression models are visualized as a scatter of the fitted values and best-fit lines. All analyses are done using R, version 4.2.2, using the “lme4”, “visreg”, and “effects” packages.

## Results

Patient characteristics

Eleven patients were recruited in the epidural group, and 18 patients in the LSPB group. The median age of the patients in the epidural group was 73.5 years (65.75-82.25), while it was 76 years (70-81) in the LSPB group. Four patients (36.4%) were males in the epidural group, while 14 patients (77.8%) were males in the LSPB group. The median height in the epidural group was 160 cm (155-165) and 162 cm (160-170) in the LSPB group. The median weight in the epidural group was 62.5 kg (58.75-84.25) and 65 kg (55-70) in the LSPB group. The median BSA in the epidural group was 1.65 m^2^ (1.58-1.86) and 1.68 m^2^ (1.56-1.81) in the LSPB group. The median BMI in the epidural group was 25.95 kg/m^2^ (23.35-27.81) and 23.67 kg/m^2^ (22.04-25.39) in the LSPB group. The demographic and other relevant details are summarized in Table 1.

**Table 1:** Demographics of patients receiving continuous lumbar epidural and lumbosacral plexus blocks

	Continuous Lumbar Epidural (CLE)	Lumbosacral Plexus Block (LSPB)
Number of Patients (n)	11	18
Median Age (Years)	73.5 (65.75-82.25)	76 (70-81)
Gender (n/%)		
Male	4 (36.4)	14 (77.8)
Female (%)	7 (63.63)	4 (22.22)
Height (cm)	160 (155-165)	162 (160-170)
Weight (kg)	62.5 (58.75-84.25)	65 (55-70)
Body Surface Area (m^2^)	1.65 (1.58-1.86)	1.68 (1.56-1.81)
Body Mass Index (kg/m^2^)	25.95 (23.35-27.81)	23.67 (22.04-25.39)

Change in vital parameters

**Figure 2:**
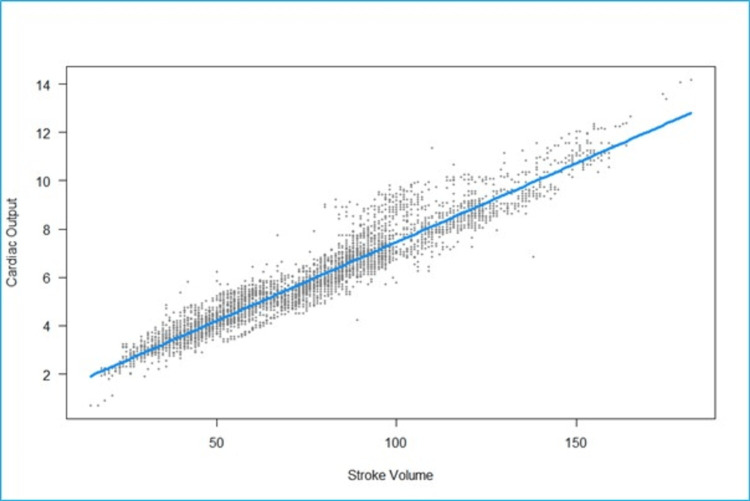
Scatterplot of fitted values of stroke volume (milliliters) vs cardiac output (liters/minute). The scatter points are tightly clustered around the regression line, indicating a high correlation.

We fitted a linear mixed model to predict CO with SV (Figure 2). The model included participants as the random effect. The model's total explanatory power is substantial (conditional R2 = 0.93), and the part related to the fixed effects alone (marginal R2) is 0.74. The effect of SV is statistically significant and positive. For each unit increase in SV, CO increases by 0.07 (95% CI: 0.06 to 0.07), p < 0.001.

**Figure 3:**
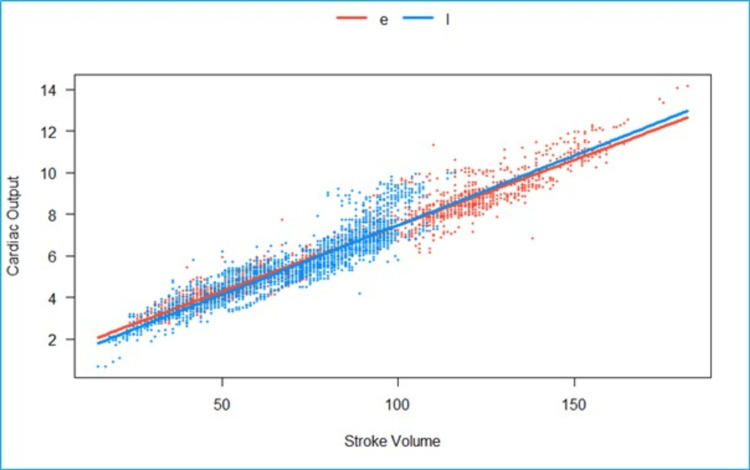
Grouped scatterplot of fitted values of stroke volume (milliliters) vs. cardiac output (liters/minute). The effect of the SV in the LSPB group is statistically significant and positive when compared to the epidural group. e- Epidural, l- lumbosacral plexus block, Scattered orange- epidural, scattered blue- Lumbosacral plexus block

We fitted a linear mixed model to predict CO with SV, grouped over the type of block (Epi vs. LSPB) (Figure 3). The model included participants as the random effect. The model’s total explanatory power is substantial (conditional R2 = 0.93), and the part related to the fixed effects alone (marginal R2) is 0.73. The effect of the SV × group (LSPB) is statistically significant and positive. Compared to group (Epi), for each unit increase in SV in the group (LSPB), the rate of increase in CO is higher by 3.6e-3 (95% CI: 1.4e-3 to 5.8e-3), p = 0.001.

**Figure 4:**
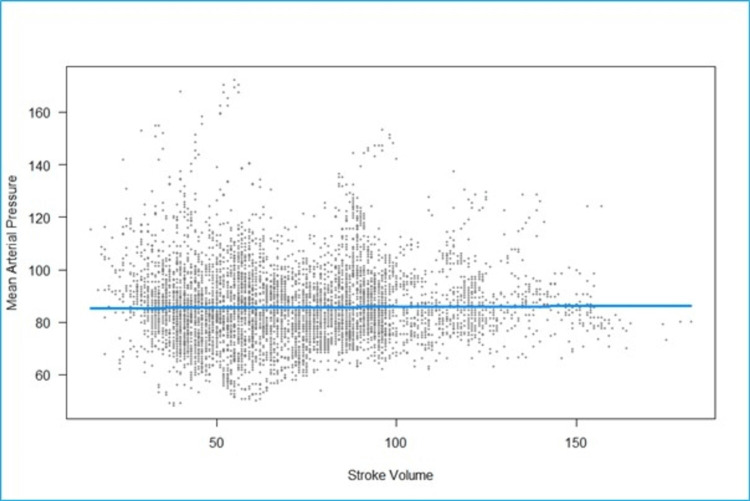
Scatterplot of fitted values of stroke volume (milliliters) vs mean arterial pressure (mmHg). The fitted regression line is nearly horizontal, indicating no significant linear relationship between stroke volume and mean arterial pressure.

We fitted a linear mixed model to predict MAP with SV (Figure 4). The model included participants as the random effect. The model's total explanatory power is substantial (conditional R2 = 0.53), and the part related to the fixed effects alone (marginal R2) is 5.79e-05. The effect of SV is statistically non-significant and positive. For each unit increase in SV, MAP increases by 0.005 (95% CI: -0.02 to 0.03), p = 0.692.

**Figure 5:**
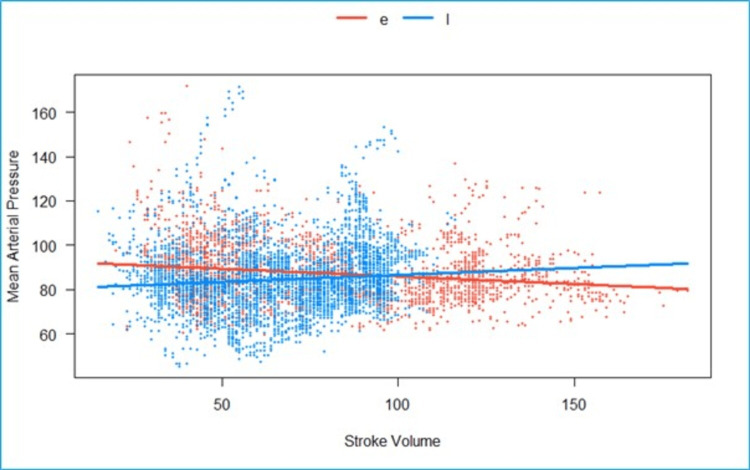
Grouped scatterplot of fitted values of stroke volume (milliliters) vs mean arterial pressure mmHg). The effect of the SV in the LSPB group is statistically significant and positive when compared to the epidural group e- Epidural, l- lumbosacral plexus block, Scattered orange- epidural, scattered blue- Lumbosacral plexus block

We fitted a linear mixed model to predict MAP with SV, grouped over the type of block (CLE vs. LSPB) (Figure 5). The model included participants as the random effect. The model’s total explanatory power is substantial (conditional R2 = 0.53), and the part related to the fixed effects alone (marginal R2) is 0.01. The effect of the SV × group (LSPB) is statistically significant and positive. Compared to group (CLE), for each unit increase in SV in the group (LSPB), the rate of increase in MAP is higher by 0.13 (95% CI: 0.07 to 0.19), p <.001.

**Figure 6:**
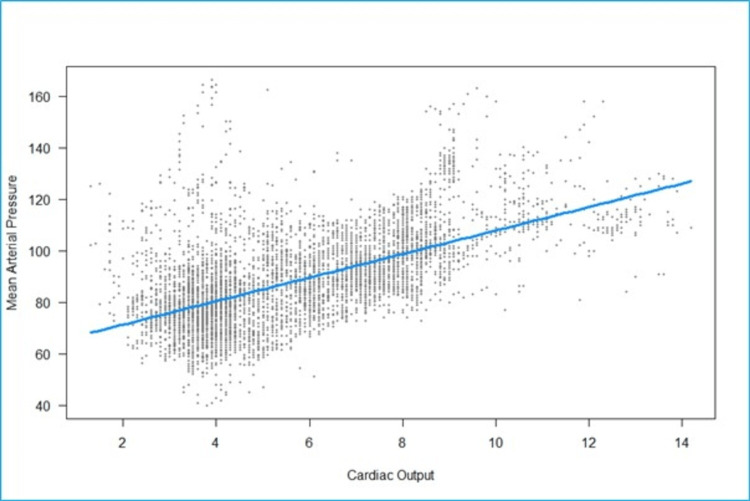
Scatterplot of fitted values of cardiac output (liters/minute) vs mean arterial pressure (mmHg). The regression line is horizontal, showing no meaningful linear relationship between cardiac output (CO) and mean arterial pressure (MAP).

We fitted a linear mixed model to predict MAP with CO (Figure 6). The model included participants as the random effect. The model's total explanatory power is substantial (conditional R2 = 0.73), and the part related to the fixed effects alone (marginal R2) is 0.15. The effect of CO is statistically significant and positive. For each unit increase in CO, MAP increases by 4.56 (95% CI: 4.22 to 4.90), p <0.001.

**Figure 7:**
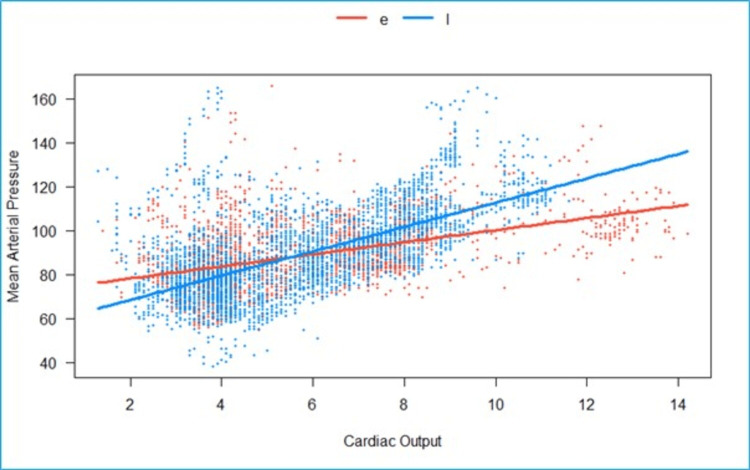
Grouped scatterplot of fitted values of cardiac output (liters/minute) vs mean arterial pressure (mmHg). The effect of CO in the LSPB group is statistically significant and positive when compared to the epidural group. e- Epidural, l- lumbosacral plexus block, Scattered orange- epidural, scattered blue- Lumbosacral plexus block

We fitted a linear mixed model to predict MAP with CO, grouped over the type of block (CLE vs. LSPB) (Figure 7). The model included participants as the random effect. The model’s total explanatory power is substantial (conditional R2 = 0.72), and the part related to the fixed effects alone (marginal R2) is 0.16. The effect of the CO × group (LSPB) is statistically significant and positive. Compared to group (Epi), for each unit increase in CO in the group (LSPB), the rate of increase in MAP is higher by 2.78 (95% CI: 2.07 to 3.50), p <0.001.

**Figure 8:**
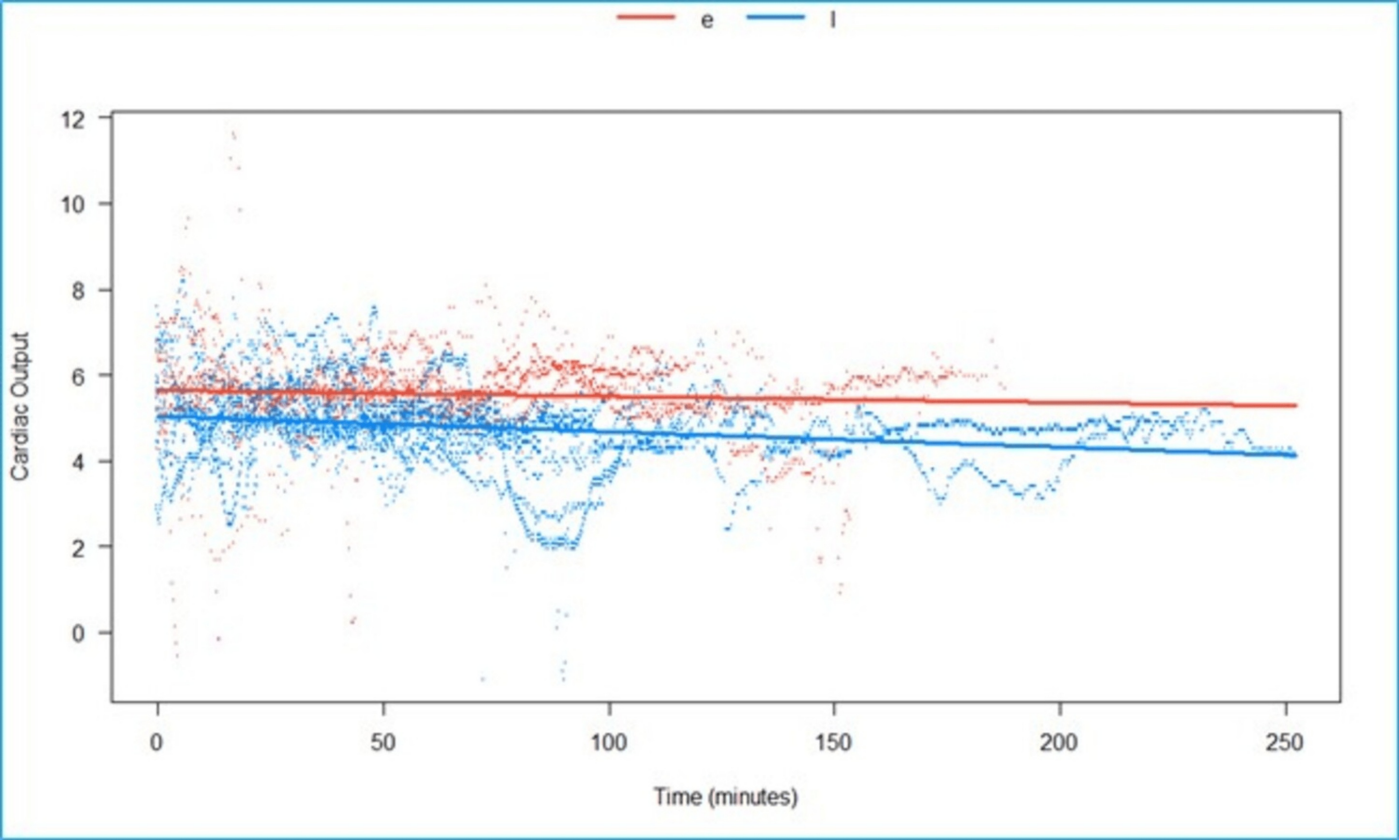
Predicted cardiac output (in liters/minute) over time, grouped by type of block. The effect of time in the LSPB group is statistically significant and negative when compared to the epidural group. e- Epidural, l- lumbosacral plexus block, Scattered orange- epidural, scattered blue- Lumbosacral plexus block

We fitted a linear mixed model to predict CO with time, grouped over the type of block (Epi vs. LSPB) (Figure 8). The model included participants as the random effect. The model’s total explanatory power is substantial (conditional R2 = 0.87), and the part related to the fixed effects alone (marginal R2) is 0.03. The effect of time × group (LSPB) is statistically significant and negative. Compared to group (Epi), for each minute passed under the block-group (LSPB), the rate of increase in CO is lower by 2.18 (95% CI: 1.22e-3 to 3.13e-3), p <0.001.

**Figure 9:**
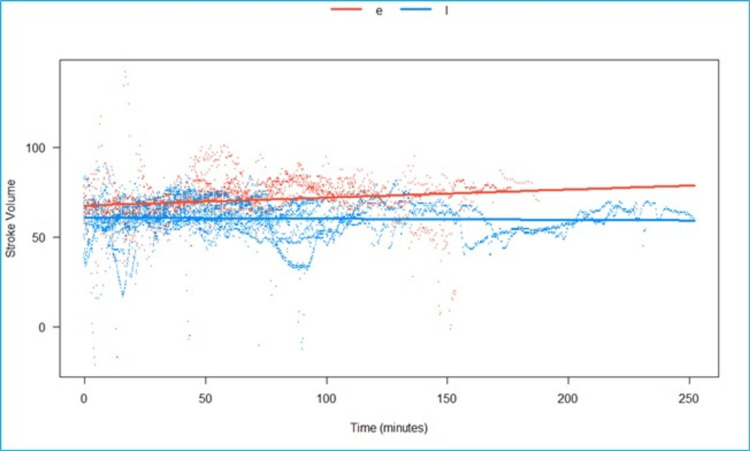
Predicted stroke volume (in milliliters) over time, grouped by type of block, is statistically significant and negative in the LSPB group when compared to the epidural group e- Epidural, l- lumbosacral plexus block, Scattered orange- epidural, scattered blue- Lumbosacral plexus block

We fitted a linear mixed model to predict SV with time, grouped over the type of block (Epi vs. LSPB) (Figure 9). The model included participants as the random effect. The model’s total explanatory power is substantial (conditional R2 = 0.88), and the part related to the fixed effects alone (marginal R2) is 0.03. The effect of time × group (LSPB) is statistically significant and negative. Compared to group (Epi), for each minute passed under the block-group (LSPB), the rate of increase in SV is lower by 0.05 (95% CI: 0.04 to 0.06), p <0.001.

**Figure 10:**
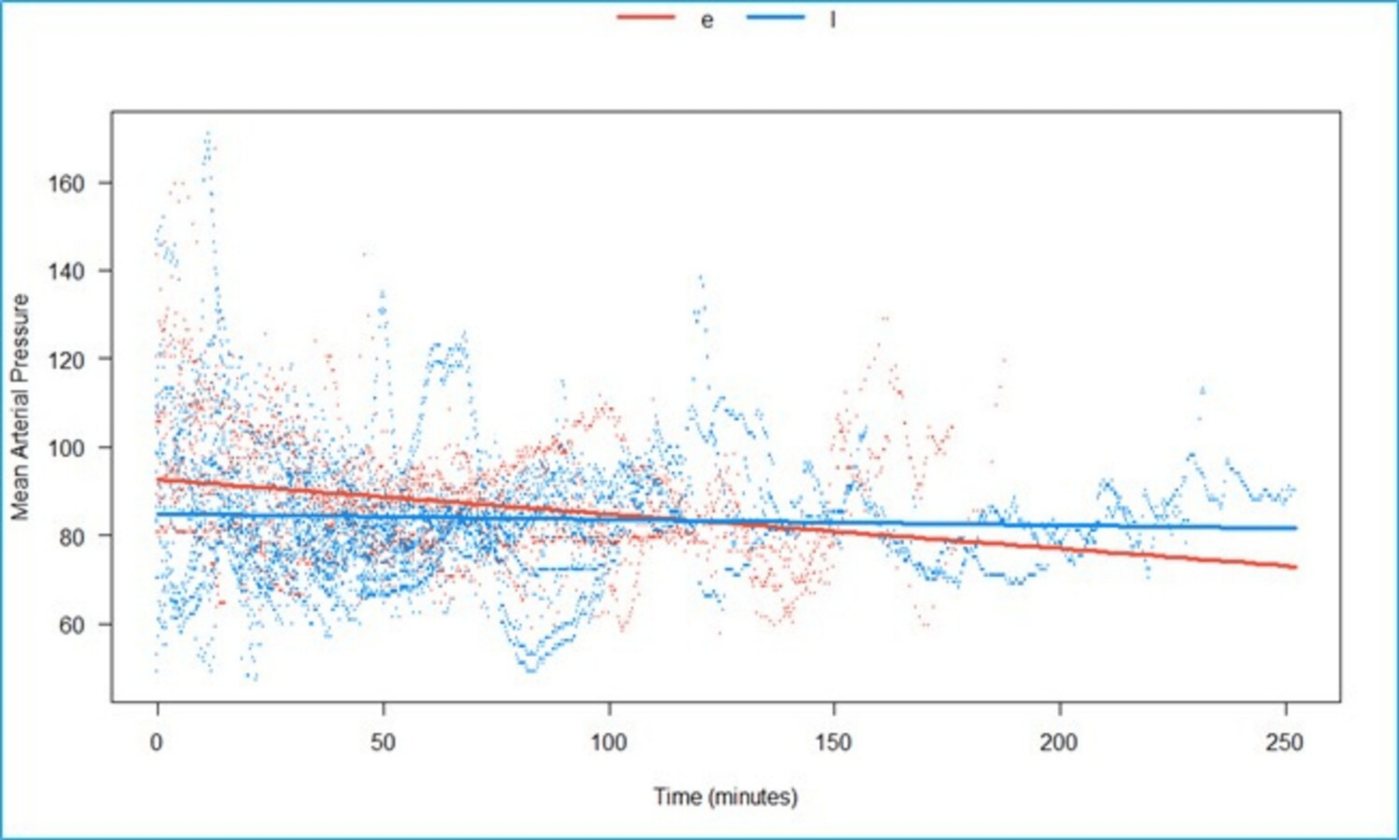
Predicted mean arterial pressure (in mmHg) over time, grouped by type of block, is statistically significant and negative in the LSPB group when compared to the epidural group e- Epidural, l- lumbosacral plexus block, Scattered orange- epidural, scattered blue- Lumbosacral plexus block

We fitted a linear mixed model to predict MAP with time, grouped over the type of block (Epi vs. LSPB) (Figure 10). The model included participants as the random effect. The model’s total explanatory power is substantial (conditional R2 = 0.53), and the part related to the fixed effects alone (marginal R2) is 0.02. The effect of time × group (LSPB) is statistically significant and negative. Compared to group (Epi), for each minute passed under the block-group (LSPB), the rate of increase in SV is higher by 0.07 (95% CI: 0.05 to 0.08), p <0.001.

Interventions

Interventions

In the entire cohort of patients, 1220 mL of crystalloids were given to patients who received LSPB, while 1700 mL of crystalloids were given to patients who received an epidural. In four patients in whom LSPB was administered, noradrenaline and vasopressors were given, while six patients in the epidural group received noradrenaline and vasopressors.

## Discussion

Approximately 50% of elderly multi-comorbid patients with PFFs are frequently admitted in a dehydrated state. Untreated or unrecognized preoperative dehydration leads to in-house complications, perioperative hypotension, and adverse postoperative outcomes. The SAB is the single most frequently implemented regional anesthesia technique in this cohort. Nevertheless, the duration of the block and the quality of surgical anesthesia were suboptimal, even though a volume of 3.5 mg bupivacaine was associated with a reduced incidence of hypotension. A mildly elevated risk was observed as a result of prolonged exposure (10 minutes) to MAP < 80mmHg. The risks increased to moderate levels when exposed to MAPs less than 60-65 mmHg for 5 minutes. However, high risks were reported for MAP less than 65 mmHg for a duration of more than 20 minutes, MAP less than 50 mmHg for 5 minutes, or any exposure <40 mmHg. Additionally, a study demonstrated the detrimental effects of SAB in elderly patients undergoing PFN, as well as the advantageous effects of segmental epidural and LSPBs [1]. In elderly multi-comorbid patients (ASA 3 and 4), our investigation employed segmental epidural and LSPBs and examined their hemodynamic effects using the FloTrac/Vigileo™ system.

In the past, two studies yielded conflicting results; one showed an increase in CO, while the other did not [7, 9]. A study contrasted the initial decrease of non-invasive MAP in the block-group (27%) to the subarachnoid bupivacaine group (38%), even though the difference was statistically insignificant. Nevertheless, the subarachnoid group experienced hypotension for a prolonged duration, necessitating frequent ephedrine doses (p<0.05). Additionally, patients who were over the age of 85 experienced a more significant decrease in their MAPs (p<0.01). [8] The FloTrac/Vigileo™ system was used to monitor hemodynamic changes in a study, which determined that the LPB did not affect the cardiac index. Although statistically significant, alterations in heart rate and arterial blood pressure remained within a clinically acceptable range (<10% variation) [7]. A unilateral sympathectomy would be the primary outcome of an LPB that is administered between the anterior 2/3 and posterior 1/3 of the psoas muscle. A dose-dependent negative ionotropic effect can be produced by the rapid absorption of a large volume of LA from this area [9]. Nevertheless, no substantial decrease in hemodynamic variables was observed, likely due to the successful implementation of a mini-fluid challenge to maintain the SV within the target range. A previous investigation [10] compared the hemodynamic effects of subarachnoid bupivacaine and alkalinized lignocaine. The CO was measured using a transthoracic bioelectrical impedance. The fall in the cardiac index was statistically significant and was attributed to bradycardia, with the SV remaining unchanged. Epidurals facilitate a progressive blockade of the sympathetic outflow, thereby enabling the compensatory mechanism to maintain a stable hemodynamics [10]. Epidural anesthesia aids in maintaining the afterload, one of the determinants of the CO, with a minimal change in the systemic vascular resistance. It is acknowledged that a decrease in systemic vascular resistance following a SAB in a cohort over the age of 80 is attributed to a decrease in CO. [11] Patients aged 65 years or older who underwent SAB for hip arthroplasty and were monitored with the LiDCOplusTM exhibited a decrease in the SV index, cardiac index, and oxygen deliver index (DO2I) both before and following the injection of spinal anesthesia. The authors caution against the correlation between the reduced SV index and post-spinal hypotension as a manifestation of pre-existing diastolic dysfunction [11].

The elderly population (ASA-PS III, IV) undergoing PFN for PFF was suitable for our study, which involves two critical regional anesthesia techniques: segmental epidural, which provides a graded block of the sympathetic outflow, and LSPB, which incriminates unilateral sympathetic outflow.

The FloTrac/Vigileo™ system necessitates the in-situ placement of a radial artery catheter (RAC). All patients with multi-comorbidities, particularly those with moderate to severe cardiac decompensation, are monitored invasively using RAC. This does not necessitate calibration, as it has been validated using a pulmonary artery catheter (PAC) [12-14]. Nevertheless, the insertion and maintenance of a PAC would pose a challenge in this cohort. The FloTrac/Vigileo™ system would offer practical information regarding the current evaluation of cardiac function, as early surgery timing is the primary factor in this cohort.

The FloTrac/Vigileo™ system enables the continuous and simultaneous monitoring of intravascular fluid management and hypotension. Minimal boluses of crystalloids are administered to prevent a decrease in preload, an additional critical variable in the maintenance of CO, through continuous monitoring of the SV. It is also beneficial to attain the targeted SV target, which is the threshold at which fluid infusions would not result in any change in the SV. Therefore, the FloTrac/Vigileo™ system is instrumental in achieving two critical objectives: the appropriate perfusion of tissue and the delivery of oxygen (with normal hemoglobin levels).

A significant limitation is the use of an observational study with a limited sample size. Fluid responsiveness in mechanically ventilated patients is primarily predicted by the SV variation [15]. In essence, we did not measure SVV because all of our patients were awake during hemodynamic monitoring. SV was employed to forecast fluid response. This could be a significant limitation. In addition, we did not assess the systemic vascular resistance, pulmonary capillary wedge pressure, or central venous pressure. The plasma levels were not measured, and epidural diffusion was not precluded, as this block was high-profile and at risk of LA toxicity. This was an observational, non-randomized, single-center study with a relatively small sample size. We acknowledge that this limits external validity. There was a significant imbalance between male and female patients. This could definitely confound the results, which is a major limitation.

## Conclusions

The FloTrac/Vigileo™ system demonstrated that both the CLE and the LSPB maintained stable hemodynamics. SV could be used to predict intravenous fluid management in addition to hemodynamics. In elderly patients with comorbidities who are undergoing surgeries for hip fractures, the use of the FloTrac/Vigileo™ system can guide hemodynamic and fluid management. These results could influence clinical decisions on the choice of anesthetic block, considering their differential effects on hemodynamic parameters. Further research may validate these findings and refine anesthetic practices to optimize patient outcomes.
